# Statistically-Estimated Tree Composition for the Northeastern United States at Euro-American Settlement

**DOI:** 10.1371/journal.pone.0150087

**Published:** 2016-02-26

**Authors:** Christopher J. Paciorek, Simon J. Goring, Andrew L. Thurman, Charles V. Cogbill, John W. Williams, David J. Mladenoff, Jody A. Peters, Jun Zhu, Jason S. McLachlan

**Affiliations:** 1 Department of Statistics, University of California, Berkeley, California, United States of America; 2 Department of Geography, University of Wisconsin, Madison, Wisconsin, United States of America; 3 VA Office of Rural Health, Veterans Rural Health Resource Center, Iowa City VAMC, Iowa City, Iowa, United States of America; 4 Harvard Forest, Harvard University, Petersham, Massachusetts, United States of America; 5 Center for Climatic Research, University of Wisconsin, Madison, Wisconsin, United States of America; 6 Department of Forest and Wildlife Ecology, University of Wisconsin, Madison, Wisconsin, United States of America; 7 Department of Biological Sciences, University of Notre Dame, Notre Dame, Indiana, United States of America; 8 Department of Statistics, University of Wisconsin, Madison, Wisconsin, United States of America; Chinese Academy of Sciences, CHINA

## Abstract

We present a gridded 8 km-resolution data product of the estimated composition of tree taxa at the time of Euro-American settlement of the northeastern United States and the statistical methodology used to produce the product from trees recorded by land surveyors. Composition is defined as the proportion of stems larger than approximately 20 cm diameter at breast height for 22 tree taxa, generally at the genus level. The data come from settlement-era public survey records that are transcribed and then aggregated spatially, giving count data. The domain is divided into two regions, eastern (Maine to Ohio) and midwestern (Indiana to Minnesota). Public Land Survey point data in the midwestern region (ca. 0.8-km resolution) are aggregated to a regular 8 km grid, while data in the eastern region, from Town Proprietor Surveys, are aggregated at the township level in irregularly-shaped local administrative units. The product is based on a Bayesian statistical model fit to the count data that estimates composition on the 8 km grid across the entire domain. The statistical model is designed to handle data from both the regular grid and the irregularly-shaped townships and allows us to estimate composition at locations with no data and to smooth over noise caused by limited counts in locations with data. Critically, the model also allows us to quantify uncertainty in our composition estimates, making the product suitable for applications employing data assimilation. We expect this data product to be useful for understanding the state of vegetation in the northeastern United States prior to large-scale Euro-American settlement. In addition to specific regional questions, the data product can also serve as a baseline against which to investigate how forests and ecosystems change after intensive settlement. The data product is being made available at the NIS data portal as version 1.0.

## Introduction

Historical datasets provide critical context to understand forest ecology. They allow researchers to define ‘baseline’ conditions for conservation management, to understand ecosystem processes at decadal and centennial scales, to track forest responses to shifting climates, and, particularly in regions with widespread land use change, to understand the extent to which forests after conversion and regeneration differ from the original forest cover.

Euro-American settlement and subsequent land use change occurred in a time-transient fashion across North America and were accompanied by land surveys needed to demarcate land for land tenure and use. Various systems were used by surveyors to locate legal boundary markers, usually by recording and marking trees adjacent to survey markers. These data provide vegetation information that can be mapped and used quantitatively to represent the period of settlement. Early surveys (from 1620 until 1825) in the northeastern United States provide spatially-aggregated data at the township level [1, 2], with typical township size on the order of 200 km^2^ and no information about the locations of individual trees; we refer to these as the Town Proprietor Survey (TPS). Later surveys after the establishment of the U.S. Public Land Survey System (PLS) by the General Land Office (GLO) provide point-level data along a regular grid, with one-half mile (800 m) spacing, for Ohio and westward during the period 1785 to 1907 [3–6]. At each point 2-4 trees were identified, and the common name, diameter at breast height, and distance and bearing from the point were recorded. Survey instructions during the PLS varied through time and by point type. Accounting for this variation requires data screening to maximize consistency among points and the application of spatially-varying correction factors [6] to accurately assess tree stem density, basal area and biomass from the early settlement records, but the impact on composition estimates is limited [7]. Surveyors sometimes used ambiguous common names, which requires matching to scientific names and standardization [6, 8].

Logging, agriculture, and land abandonment have left an indelible mark on forests in the northeastern United States [2, 6, 9, 10]. However most studies have assessed these effects in individual states or smaller domains [11, 12] and with various spatial resolutions, from townships (36 square miles) to forest zones of hundreds or thousands of square miles. [6] provide a new dataset of forest composition, biomass, and stem density based on PLS data for the upper Midwest that is resolved to an 8 km by 8 km grid cell scale, providing broad spatial coverage at a spatial scale that can be compared to modern forests using Forest Inventory and Analysis products [13]. Combined with additional, coarsely-sampled PLS data from Illinois and Indiana, newly-digitized data from southern Michigan, and with the TPS data, this gives us raw data for much of the northeastern United States. However, there are several limitations of using the raw data that can be alleviated by the use of a statistical model to develop a statistically-estimated data product. First, the PLS and TPS data only provide estimates of within-cell variance that do not account for information from nearby locations. Second, there are data gaps: the available digitized data from Illinois and Indiana represent a small fraction of those states, and missing townships are common in the TPS data. Third, the TPS and PLS data have fundamentally different sampling design and spatial resolution. Our statistical model allows us to provide a spatially-complete data product of settlement-era tree composition for a common 8 km grid with uncertainty across the northeastern U.S.

In *Data* we describe the data sources, while in *Statistical model* we describe the statistical model used to create the data product. In *Model comparison* we quantitatively compare competing statistical specifications, and in *Data Product* we describe the final data product. In *Discussion* we discuss the uncertainties estimated by and the limitations of the statistical model, and we list related data products under development.

## Methods

### Data

**Fig 1:**
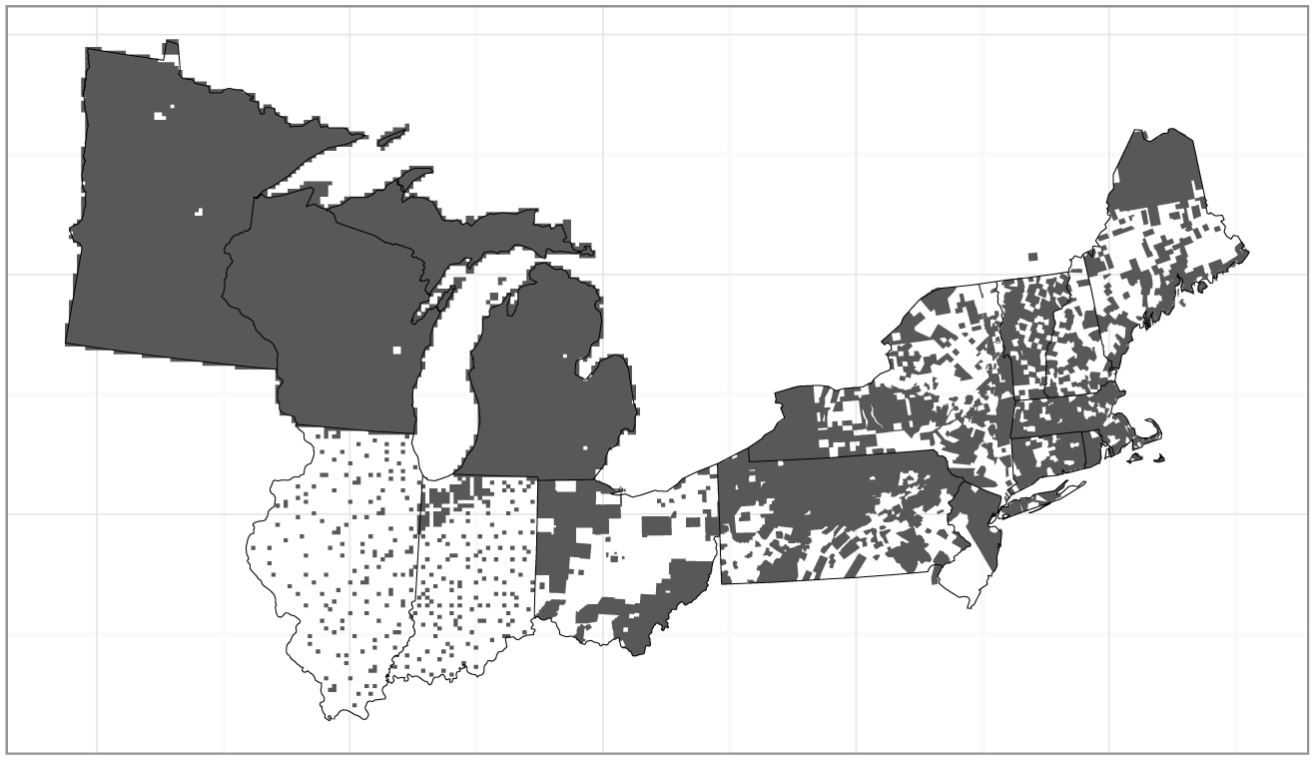
Spatial domain of the northeastern United States, with locations with data shown in gray. Locations are grid cells in midwestern portion and townships in eastern portion. In addition to locations without data being indicated in white, grid cells completely covered in water are white (e.g., a few locations in the northwestern portion of the domain in the states of Minnesota and Wisconsin).

The raw data were obtained from land division survey records collated and digitized from across the northeastern U.S. by a number of researchers (Fig 1). For the states of Minnesota, Wisconsin, Illinois, Indiana, and Michigan (the midwestern subdomain), digitized data are available at PLS survey point locations and have been aggregated to a regular 8 km grid in the Albers projection. (Note that for Indiana and Illinois, at the moment trees are associated with township centroids and then assigned to 8 km grid cells based on the centroid, but in the near future we will have point locations available for each tree.) For the states of Ohio, Pennsylvania, New Jersey, New York and the six New England states (the eastern subdomain), data are aggregated at the township level. We make predictions for all of the states listed above; these constitute our core domain. There are also data from a single township in Quebec and a single township in northern Delaware; these data help inform predictions in nearby locations within our core domain, but predictions are not made for Quebec or Delaware. Digitization of PLS data in Minnesota, Wisconsin and Michigan is essentially complete, with PLS data for nearly all 8 km grid cells, but data in Illinois and Indiana represent a sample of the full set of grid cells, with survey record transcription ongoing. Data for the eastern states are available for a subset of the full set of townships covering the domain; the TPS data for some townships were lost, incomplete, or have not been located [1].

Note that surveys occurred over a period of more than 200 years as European colonists (before U.S. independence) and the United States settled what is now the northeastern and midwestern United States. Our estimates are for the period of settlement represented by the survey data and therefore are time-transgressive; they do not represent any single point in time across the domain, but rather the state of the landscape at the time just prior to widespread Euro-American settlement and land use [1, 14]. These forest composition datasets do include the effects of Native American land use and early Euro-American settlement activities, e.g. [15], but it is likely that the imprint of this earlier land use is highly concentrated rather than spatially extensive [16].

Extensive details on the upper Midwest (Minnesota, Wisconsin, Michigan) data and processing steps are available [6]; key elements include the use of only corner points, the use of only the two closest trees at each corner point, spatially-varying correction factors for sampling effort, and a standardized taxonomy table. The lower Midwest (Illinois, Indiana) data were purchased from the Indiana State Archives (Indiana) and Hubtack Document Resources (hubtack.com; Illinois) and processed using similar steps as for the upper Midwest data. Digitization of the Illinois and Indiana data is still underway, so many grid cells contained no data at the time the statistical model was fit. Note that the number of trees per grid cell varies depending on the number of survey points in a cell, with an average of 124 trees per cell. The gridded data at the 8 km resolution for the midwest subdomain are available through the NIS data portal [17]. The TPS data were compiled by C.V. Cogbill from a myriad of archival sources representing land division surveys conducted in connection with local settlement and are available through the NIS data portal [18, 19].

The aggregation into taxonomic groups is primarily at the genus level but is at the species level in some cases of monospecific genera. We model the following 22 taxa plus an “other hardwood” category: Atlantic white cedar (*Chamaecyparis thyoides*), Ash (*Fraxinus spp.*), Basswood (*Tilia americana*), Beech (*Fagus grandifolia)*, Birch (*Betula spp.*), Black gum/sweet gum (*Nyssa sylvatica* and *Liquidambar styraciflua*), Cedar/juniper (*Juniperus virginiana* and *Thuja occidentalis*), Cherry (*Prunus spp.*), Chestnut (*Castanea dentata*), Dogwood (*Cornus spp.*), Elm (*Ulmus spp.*), Fir (*Abies balsamea*), Hemlock (*Tsuga canadensis*), Hickory (*Carya spp.*), Ironwood (*Carpinus caroliniana* and *Ostrya virginiana*), Maple (*Acer spp.*), Oak (*Quercus spp.*), Pine (*Pinus spp.*), Poplar/tulip poplar (*Populus spp.* and *Liriodendron tulipifera*), Spruce (*Picea spp.*), Tamarack (*Larix laricina*), and Walnut (*Juglans spp.*). Note that in several cases (black gum/sweet gum, ironwood, poplar/tulip poplar, cedar/juniper), because of ambiguity in the common tree names used by surveyors, a group represents trees from different genera or even families. For the midwestern subdomain we do not fit statistical models for Atlantic white cedar and chestnut as these species have 0 and 7 trees present, respectively. The taxa grouped into the other hardwood category are those for which fewer than roughly 2000 trees were present in the dataset; however, we include Atlantic white cedar explicitly despite it only having 336 trees in the dataset because of specific ecological interest in Atlantic white cedar wetlands.

Diameters are only recorded in the PLS data. Although surveyors avoided using small trees, there was no consistent lower diameter limit. The PLS data generally represent trees greater than 8 inches (ca. 20 cm) diameter at breast height (dbh), but with some trees as small as 1 inch dbh (smaller trees were much more common in far northern Minnesota). TPS data have no information about dbh, but the trees were large enough to blaze and are presumed to be relatively large trees useful for marking property boundaries.

There are approximately 860,000 trees in the midwestern subdomain and 420,000 trees in the eastern subdomain. In the midwestern subdomain, oak is the most common taxon and pine the second most common, while in the eastern subdomain oak is the most common and beech the second most common.

Our domain is a rectangle covering all of the states using a metric Albers (Great Lakes and St. Lawrence) projection (PROJ4: EPSG:3175), with the rectangle split into 8 km cells, arranged in a 296 by 180 grid of cells, with the centroid of the cell in the southwest corner located at (-71000 m, 58000 m). For the midwestern subdomain we use the western-most 146 by 180 grid of cells when fitting the statistical models. For the eastern subdomain we use the eastern-most 180 by 180 grid of cells and then omit 23 rows of cells in the north and 17 rows of cells in the south, as these grid cells are outside of the states containing data.

### Statistical model

We fit a Bayesian statistical model to the data, with two primary goals:

1. To estimate composition on a regular grid across the entire domain, filling gaps where no data are available, and
2. To quantify uncertainty in composition at all locations. Even in grid cells and townships with data, we wish to quantify uncertainty because the empirical proportions represent estimates of the true proportions that could be calculated using the full population of all the trees in a grid cell or township.

At a high level, the Bayesian statistical model estimates composition across the domain, even in locations with sparse or no data, by combining the raw composition data with the assumption that composition varies in a smooth spatial fashion across the domain. The information in the data is quantified by the data model, also known as the likelihood. The assumption of smoothness is built into the model by representing the true unknown spatially-varying composition using a statistical spatial process representation that induces smoothing of estimates across nearby locations. This spatial process representation is a form of prior distribution and is a function of model parameters called hyperparameters that determine the correlation structure of the process and are also estimated based on the data.

The result of fitting the Bayesian model via Markov chain Monte Carlo (MCMC) is a set of representative samples from the posterior distribution for the composition in the 23 taxonomic groupings at each of the grid cells. These samples are the data product (described further in *Data Product*) and can be used in subsequent analyses. The mean and standard deviation of the samples for each pair of cell and taxon represent our best estimate (i.e., prediction) of composition and a Bayesian “standard error” quantifying the uncertainty in the estimate.

In the remainder of this section we provide the technical specification of the model and of the computations involved in fitting the model.

#### Data model

We start by describing the basic model for those states for which we have raw data on the 8 km grid, and in *Model for township data* we describe the extension of the model to accommodate data aggregated at the township level.

The statistical model treats the observations as coming from a multinomial distribution with a (latent) vector of proportions for each grid cell,

$$\begin{array}{c} y_{i}∼\text{Multi}\left(n_{i},\theta \left(s_{i}\right)\right), \end{array}$$

where *y*_*i*_ is the vector of counts for the *P* taxa at the *i*th cell, *n*_*i*_ is the number of trees counted in the cell, and *θ*(*s*_*i*_) is the vector of unknown proportions for those taxa at that cell. Note that we use a standard multinomial distribution without overdispersion, because the set of trees in the dataset is roughly uniformly sampled across the cells or townships [6].

The proportions, *θ*_*p*_(*s*_*i*_), *p* = 1, …, *P*, are modeled spatially by a set of *P* Gaussian spatial processes, one per taxon, *α*_*p*_(*s*_*i*_), *p* = 1, …, *P*. This collection of processes defines a multivariate spatial process for composition. The *α*_*p*_(*s*) processes are defined on the 8 km grid, *α*_*p*_ = {*α*_*p*_(*s*_1_), …, *α*_*p*_(*s*_*m*_)} for the *m* grid cells. In *Latent variable model* we introduce a multinomial probit model that relates the *α*_*p*_(*s*) processes to the proportion processes, *θ*_*p*_(*s*), via the introduction of latent variables, with an implicit sum-to-one constraint, $\sum_{p=1}^{P}\theta_{p}\left(s\right)=1$. A multinomial probit model is similar to logistic regression, used for modeling a binary outcome based on an underlying probability the outcome will occur, but generalizes to modeling a categorical variable based on probabilities for each category.

The critical component of the statistical model is the representation of *α*_*p*_(*s*) as a spatial process. A spatial process is a statistical representation that models spatially-correlated values. It provides a prior structure that serves to smooth across noise in the observations and allows for prediction at locations based on information from nearby locations, including interpolation to locations with no data. Apart from the sum-to-one constraint, the taxa are considered to be independent in the prior. We did not want to impose any structure that ties the different taxa together, as any correlation will likely vary across space.

In the next section, we consider two spatial models to define the structure of the *α*_*p*_(*s*) processes, a standard conditional autoregressive model [20] and a Gaussian Markov random field (MRF) approximation to a Gaussian process with Matérn covariance [21]. These models are specific statistical formulations of spatial processes that represent spatial correlation by defining neighborhoods around each location that are used to help inform predictions at the location.

#### Spatial process models

MRF models represent the neighborhood information by working directly with the precision matrix (the inverse of the covariance matrix) of the values of the spatial process, so calculation of the prior density of *α*_*p*_ is computationally simple [22]. However, in situations in which the likelihood is not normal, such as our multinomial likelihood, it can be difficult to set up effective MCMC algorithms that are able to move in the high-dimensional space of *α*_*p*_. The latent variable representation helps to alleviate this problem. Next we describe two alternative spatial models that we considered; in *Model comparison*, we evaluate the models on held-out data to choose between the two.

#### Standard conditional autoregressive models

Our first model is a standard conditional autoregressive (CAR) model; technical details can be found in [20]. We use a standard form of this model that treats the four cardinal neighbors of each grid cell as the neighbors of the grid cell. The corresponding precision matrix has diagonal elements, *Q*_*ii*_, equal to the number of neighbors for the *i*th area (i.e., four except for cells on the boundary of the domain), while *Q*_*ik*_ = −1 (the negative of a weight of one) when areas *i* and *k* are neighbors and *Q*_*ik*_ = 0 when they are not. This gives the following model for the values of *α*_*p*_(*s*_*i*_) collected as a vector across all of the grid cells, *i* = 1, …, *m*:

$$\begin{array}{c} \alpha_{p}∼\text{N}\left(0,\sigma_{p}^{2}Q^{-}\right). \end{array}$$

The use of the generalized inverse notation indicates that *Q* is not full-rank, but is of rank *m* − 1; this gives an improper prior on an implicit overall mean for the process values. Note that we specify an explicit mean of zero because a non-zero mean would not be identifiable in light of the implicit mean. This specification is called an *intrinsic conditional autoregression (ICAR)* and we can write *Q* = *D* − *C* where *C* is the *m* × *m* adjacency matrix defining the neighborhood relation of the locations; that is *C*_*ik*_ = 1 if locations *i* and *k* are neighbors and zero otherwise. The matrix *D* is an *m* × *m* diagonal matrix containing the row sums of matrix *C* as the diagonal entries, $D{}_{ii}=\sum_{k=1}^{m}C{}_{ik}.$

We refer to this as the *CAR model*.

#### Gaussian process approximation

Gaussian processes (GP) are also standard models for spatial processes [20]. GP models are computationally challenging for large datasets because of computational manipulations involving large covariance matrices. Given this, [21] proposed a new framework for using Gaussian MRFs (GMRFs) as approximations to GPs, based on the use of stochastic partial differential equations (SPDEs).

Gaussian processes are generally constructed using one of a number of correlation functions that define how the strength of correlation between the values of the process at two locations decays as a function of the distance between the locations. We consider Gaussian processes in the commonly-used Matérn class, using the following parameterization of the Matérn correlation function,

$$\begin{array}{c} R\left(d\right)=\frac{1}{\Gamma \left(\nu \right)2^{\nu -1}}{\left(\frac{2\sqrt{\nu }d}{\rho }\right)}^{\nu }K_{\nu }\left(\frac{2\sqrt{\nu }d}{\rho }\right), \end{array}$$

where *d* is Euclidean distance, *ρ* is the spatial range parameter, and $K_{\nu }\left(\cdot \right)$ is the modified Bessel function of the second kind, whose order is the smoothness (differentiability) parameter, *ν* > 0. *ν* = 0.5 gives the exponential covariance. For any pair of locations, *R*(*d*) defines the correlation of the process, (i.e., *α*_*p*_(*s*) in our context), as a function of the distance between the locations. Considering all pairs of locations, this defines a correlation matrix for all locations of interest.

The approach of [21] allows us to consider MRF approximations to the Matérn -based GP for *ν* = 1 and *ν* = 2. Our second spatial model is this Lindgren approximation for Matérn -based GPs with *ν* = 1. To implement this Lindgren model, one modifies the *Q* matrix defined previously as follows based on the technical specification of the precision matrix provided in [21]. Let $a=4+\frac{1}{\rho^{2}}$. The diagonal elements of *Q* are 4 + *a*^2^. The entries corresponding to cardinal neighbors are −2*a*. Those for diagonal neighbors are 2, and those for 2nd-order cardinal neighbors are 1. This extends the neighborhood structure relative to the CAR model and parameterizes it as a function of *ρ*.

The primary difference between the CAR and Lindgren models is that the Lindgren model provides an additional degree of freedom by estimating *ρ*. In particular *ρ* allows us to estimate the locality of the spatial smoothing. As *ρ* decreases, the model uses increasingly localized data to estimate the compositional proportions at a given location, effectively averaging the empirical proportions over smaller neighborhoods. In general, the [21] model will generally provide for a smoother estimate than the CAR model [23].

To ensure that the *σ*^2^ parameter is mathematically equivalent between the two models, we reparameterize, producing our second model:

$$\begin{array}{c} \alpha_{p}∼\text{N}\left(\mu_{p},\sigma_{p}^{2}\cdot \frac{4\pi }{\rho_{p}^{2}}Q{\left(\rho_{p}\right)}^{-1}\right) \end{array}$$

We refer to this model as the *SPDE model*.

#### Prior distributions

The ICAR specification contains a set of hyperparameters $\{\sigma_{p}^{2}\},$ *p* = 1, …*P*. Following [24] we use a uniform distribution on each *σ*_*p*_ parameter, with upper bound of 1000. For the SPDE model we also have hyperparameters {*μ*_*p*_}, which we give flat, non-informative priors (truncated at ±10), and {*ρ*_*p*_}, which we give uniform priors on the interval (0.1, exp(5)). These various hyperparameters are unknown parameters that control the spatial structure of the two spatial models and are estimated from both the data and the prior distributions just specified based on the Bayesian approach.

#### Latent variable model

It is well-known that devising an effective MCMC algorithm for models with latent Gaussian process(es) and a non-Gaussian likelihood is difficult [22, 25, 26]. To develop an algorithm, we make use of a latent variable representation for the multinomial probit model [27]. The representation introduces latent variables that allow one to develop an MCMC sampling strategy that takes advantage of closed-form full conditional distributions (so-called Gibbs sampling steps) for *α*_*p*_.

Suppose that compositional counts are available at a number of locations. At location *i*, a sample of size *n*_*i*_ observations is collected, and each observation (i.e., each tree) can be classified into *P* distinct categories. For a given tree *j* at location *i*, let *Y*_*ij*_ denote the response variable indicating the category. Let *Y*_*ij*_ be associated with *P* latent variables *W*_*ij*1_, …, *W*_*ijP*_ such that *Y*_*ij*_ = *p* if and only if $W_{ijp}=\max_{p^{\prime }}\{W_{ijp^{\prime }}\}$; in other words, the maximum of the set of latent variables $\left\{W_{ijp}\right\}{}_{p=1}^{P}$ determines the category of observation *j* at location *i*. The final piece of the latent variable representation is the relationship between the *W* variables and the *α*_*p*_(*s*) processes. We have that

$$\begin{array}{c} W_{ijp}∼\text{N}\left(\alpha_{p}\left(s_{i}\right),1\right) \end{array}$$

independently for all of the *W*_*ijp*_ values. Consider the following example with two locations that are neighbors and *P* = 2 categories. Each tree *j* at location *i* is associated with two variables *W*_*ij*1_ and *W*_*ij*2_, governed by the latent variables *α*_1_(*s*_*i*_) and *α*_2_(*s*_*i*_), respectively. Suppose that *α*_1_(*s*_*i*_)>*α*_2_(*s*_*i*_) for a given location *i*. Then this model implies that any tree *j* is more likely to be labeled 1 than 2 at location *i*. The difference between *α*_1_(*s*_*i*_) and *α*_2_(*s*_*i*_) explains the *difference* in probability of *categories* 1 and 2 at location *i*, and the similarity between *α*_*p*_(*s*_1_) and *α*_*p*_(*s*_2_) explains the *correlation* between the probabilities at *locations* 1 and 2 for category *p*.

#### Model for township data

We developed an extension of the model described in previous sections to account for data at a different aggregation than our core 8 km grid. This extension introduces a new set of latent variables, one per tree, that indicate the grid cells in which the trees are located and that can be sampled within the MCMC as additional unknown parameters. Specifically, *c*_*tj*_ is the latent “membership” variable for tree *j* in township *t*, *t* = 1, …, *T*. The prior for *c*_*tj*_ is a discrete distribution that puts mass, *ψ*_*ti*_, *i* = 1, …, *m*, proportional to the areal overlap between the township in which the tree is located and the *m* grid cells, giving

$$\begin{array}{c} c_{tj}∼\text{Multinom}\left(1,\left\{\psi_{t1},\ldots ,\psi_{tm}\right\}\right), \end{array}$$

independently across all trees. Because the townships overlap a limited number of grid cells, most of the *ψ*_*t*1_, …, *ψ*_*tm*_ values are zero.

Using the latent variable representation, we have that *W*_*tjp*_ ∼ N(*α*_*p*_(*s*_*c*_*tj*__), 1) for tree *j* in township *t*. In updating the other parameters in the model during the MCMC (specifically the *α* values), we condition on the current values, {*c*_*tj*_}, which provides a “soft” (i.e., probabilistic) assignment of trees to grid cells that respects both the known township in which the trees occurred and the uncertainty in which grid cells the trees occurred.

Note that this prior represents the location of each tree in a township as being independent of the other trees; this is somewhat unrealistic because it does not represent our knowledge that the trees in a township would be distributed somewhat regularly across the area of the township because the witness trees were used to indicate property boundaries.

#### Computation

The [27] representation is convenient for MCMC sampling, particularly in this high-dimensional spatial context, as it allows us to draw from the posterior conditional distributions of the *W*_*ijp*_ variables (these distributions are truncated normal) in closed form and to draw the entire vector of latent process values for each taxon, *α*_*p*_, as a single sample that respects the spatial dependence structure for each taxon.

While the latent variable representation provides great advantages in the MCMC sampling for each *α*_*p*_ compared to joint Metropolis updates or updating each location individually, there is still strong dependence between the hyperparameters, $\left\{\sigma_{p}^{2},\mu_{p},\rho_{p}\right\}$ and the latent process values (as well as between the latent process values and the latent *W*_*ijp*_ variables). To address the first, we developed a “cross-level” joint updating strategy for the CAR model in which we propose *ϕ*_*p*_ = *σ*_*p*_, *p* = 1, …, *P*, (and for the SPDE model, *ϕ*_*p*_ ∈ {*μ*_*p*_, (*σ*_*p*_, *ρ*_*p*_)}) via a Metropolis-style random walk and then given the proposed value, $\phi_{p}^{*}$, propose *α*_*p*_ from its full conditional distribution given $\phi_{p}^{*}$ and the latent *W*_*p*_ variables, where *W*_*p*_ is the vector of all *W*_*ijp*_ values for taxon *p*: *W*_*p*_ = {*W*_*ijp*_}, *i* = 1, …, *m*;*j* = 1, …, *n*_*i*_. This is equivalent to sampling from the marginalized (with respect to *α*_*p*_) distribution of *ϕ*_*p*_ conditional on *W*_*p*_. For these various joint samples of hyperparameters and *α*_*p*_, we use adaptive Metropolis sampling [28].

The full description of the MCMC sampling steps is provided in Appendix. In addition, in the latent variable representation, *θ*_*p*_(*s*) never appears explicitly and cannot be calculated in close form. Instead we use Monte Carlo integration over *W*_*ijp*_, *p* = 1, …, *P* to estimate *θ*_*p*_(*s*_*i*_), also described in Appendix.

The model is implemented in R [29] with core computational calculations coded in C++ using the *Rcpp* package [30]. We also make extensive use of sparse matrix representations and algorithms, using the *spam* package in R [31]. All code is available on Github, including pre- and post-processing code, at https://github.com/PalEON-Project/composition.

### Model comparison

#### Design

We compared the CAR and SPDE models by holding out data from the fitting process and assessing the fit of the model on the held-out data. We used two experiments with held-out data:

1. The first experiment used a subregion containing most of Minnesota and a small amount of western Wisconsin, defined to be the cells whose x-coordinate was less than 300,000 m (this defines a north-south line that approximately goes through Duluth, Minnesota) and hereafter referred to as the “Minnesota subregion”. We chose this subregion for evaluation because of its high data density, allowing us to experiment with the effects of increasing data sparsity on model performance. We held out all data from 95% of the cells in this Minnesota subregion, with cells selected at random. This was meant to assess the ability of the model to interpolate from a sparse set of cells/townships and mimics the limited data in Illinois and Indiana.
2. We held out 5% of the trees from all of the trees in the dataset for the midwestern subdomain (leaving aside the held-out Minnesota subregion cells). This was meant to assess the ability of the model to estimate the composition in cells in which data were available.

Finally, in a separate sensitivity analysis we instead left out 80% of the cells in Minnesota subregion at random. This variation on the first experiment above was meant to indicate whether our model comparison conclusions would be robust as the digitization process for Illinois and Indiana progresses and provides us with increasingly dense data.

There has been extensive work in the statistical literature on good metrics to use to compare the predictive ability of models; these metrics are referred to as scoring rules. A general conclusion from this work is that predictive distributions should maximize sharpness subject to calibration. That is, the predictive distribution should be as narrow as possible while being calibrated such that the observations are consistent with the distribution [32]. When thinking in terms of prediction intervals as summaries of the predictive distribution, we seek intervals that are as narrow as possible while still covering the truth the expected proportion (e.g., 95% for a 95% prediction interval) of the time.

Following the suggestions in [32], we considered the following metrics: Brier score, log predictive density, mean square prediction error, mean absolute error, and coverage and length of prediction intervals. Further details on each are given below. For experiment 1, we define *Y*_*i*_ = {*Y*_*i*1_, …, *Y*_*iP*_} as the count of all trees in held-out cell *i* and for experiment 2, *Y*_*i*_ is the count of held-out individual trees in the cell, while *y*_*ijp*_ is an indicator variable taking value either 0 or 1 depending on whether the *j*th held-out tree in the *i*th cell is of taxon *p*. ${\hat{\theta }}_{ip}=Y_{ip}/n_{i}$ is the empirical proportion in category *p* for the *n*_*i*_ held-out trees in cell *i*. We calculated each of the metrics in two ways. First, we used the posterior mean composition estimates (as an evaluation of our core predictions), with ${\tilde{\theta }}_{p}\left(s\right)$ being the posterior mean. Second, we averaged the metric over the posterior samples (as an evaluation of our full data product, including uncertainty), taking ${\tilde{\theta }}_{p}\left(s\right)$ to be an individual MCMC sample and then averaging the metric over all the posterior samples.

1. Brier score: [32] suggest this metric, which has been in use for decades. For multi-category as opposed to binary outcomes, this takes the form
  $$\begin{array}{c} \frac{1}{n}\sum_{i=1}^{m}\sum_{j=1}^{n_{i}}\sum_{p=1}^{P}{\left(y_{ijp}-{\tilde{\theta }}_{p}\left(s_{i}\right)\right)}^{2} \end{array}$$
  where $n=\sum_{i=1}^{m}n_{i}$ is the total number of held-out trees for a given experiment and *j* indexes across held-out trees in cell *i*.
2. Log predictive density: This metric takes the log of the probability density of held-out observations under the fitted model, $Y_{i}∼\text{Multinom}\left(n_{i},\left\{{\tilde{\theta }}_{1}\left(s_{i}\right),\ldots ,{\tilde{\theta }}_{P}\left(s_{i}\right)\right\}\right)$, summing on the log scale across all of the held-out data.
  While in principle, this metric should be optimal [33], it is very sensitive to small predictions near zero [32]. Even worse, our Monte Carlo estimation of *θ* used 10000 samples, so in some cases ${\tilde{\theta }}_{p}\left(s\right)=0$. When a tree is present in a cell but its corresponding proportion is 0, this gives a log density of −∞, preventing use of the metric. As an informal solution to this we set ${\tilde{\theta }}_{p}\left(s\right)=\frac{1}{100000}$ in such cases, but given these issues we treat the log predictive density as a secondary measure.
3. (Experiment 1 only) Weighted root mean square prediction error (RMSPE),
  $$\begin{array}{c} \sqrt{\frac{1}{Pn}\sum_{i=1}^{m}\sum_{p=1}^{P}n_{i}{\left({\hat{\theta }}_{ip}-{\tilde{\theta }}_{p}\left(s\right)\right)}^{2}} \end{array}$$
  and mean absolute error (MAE)
  $$\begin{array}{c} \frac{1}{Pn}\sum_{i=1}^{m}\sum_{p=1}^{P}n_{i}\left|{\hat{\theta }}_{ip}-{\tilde{\theta }}_{p}\left(s\right)\right|: \end{array}$$
  These metrics calculate the error of the estimated proportions relative to the empirical proportions based on the held-out trees, averaging over cells and taxa. We weight by the number of held-out trees in each cell to account for the greater variability in the empirical proportions in locations with few held-out trees.
4. (Experiment 1 only) Coverage and length of 95% prediction intervals for *Y*_*ip*_. We considered only cells with at least 50 trees to focus our assessment on cases where empirical proportions were reasonably certain and avoid being strongly influenced by predictive inference for cells where observational variability dominates.

Note that all of the metrics except coverage and interval length can be applied to individual posterior samples and therefore allow us to estimate the posterior probability that one model has a lower (better) value of the metric than the other model by simply calculating the proportion of samples for which the model has a lower value of the metric. Also note that in addition to allowing comparison between models the MAE and RMSPE metrics allow one to assess absolute performance of each model in predicting composition.

In our initial exploratory fitting, we noticed that the SPDE model produced boundary effects in the predicted composition near the edges of the convex hull of the observations. To attempt to alleviate this, we added a buffer zone with a width of six grid cells around our entire original domain, but note that the boundary effects were still evident even after inclusion of the buffer. For the model comparison, we included this buffer for both the SPDE and CAR models.

We ran each model for 150,000 iterations. After discarding 25,000 iterations for burn-in, we retained a posterior sample of 250 subsampled iterations—we use a subsample instead of the full 125,000 post-burn-in iterations to reduce post-processing computations and storage needs.

#### Results

Here we summarize the results of our analyses that inform the choice between the CAR and SPDE models.

For Experiment 1 (full cells held out) for cells in the Minnesota subregion held out of the fitting process, the CAR model outperforms the SPDE model based on the posterior distribution over the predictive metric values (Table 1). For the posterior mean predictions, the SPDE model appears to outperform the CAR model to a lesser degree, but we do not have any uncertainty estimates for this comparison. Coverage and interval lengths are similar between the two models (Table 2). From a practical perspective, based on the difference in mean absolute error, the differences between the models are small (Table 1).

**Table 1:** Predictive ability based on several predictive metric criteria for the CAR and SPDE spatial models when holding out 95% of entire cells of data in Minnesota.

	Posterior mean of metric			Metric of posterior mean predictions	
	CAR model	SPDE model	Posterior Prob. CAR <SPDE	CAR model	SPDE model
Brier	0.819	0.844	0.98	0.738	0.733
Negative Log Density	466325	510383	1.00	394003	394554
Mean Absolute Error	0.0364	0.0383	0.98	0.0275	0.0269
Root Mean Square Error	0.0897	0.0960	0.97	0.0647	0.0627

Smaller values are better for all metrics.

**Table 2:** Coverage and length of prediction intervals for the CAR and SPDE spatial models when holding out 95% of entire cells of data in Minnesota.

	CAR model	SPDE model
Coverage	0.977	0.978
Mean Interval Length	0.129	0.142
Median Interval Length	0.037	0.033

Coverage values near 0.95 are optimal, while shorter intervals are better.

The results for the variation on Experiment 1 in which the proportion of cells that are held out decreases from 95% to 80% show that the SPDE model generally outperforms the CAR model, but again differences from a practical perspective, based on mean absolute error, are limited (Tables 3 and 4).

**Table 3:** Predictive ability based on several predictive metric criteria for the CAR and SPDE spatial models when holding out 80% of entire cells of data in Minnesota.

	Posterior mean of score			Score of posterior mean predictions	
	CAR model	SPDE model	Posterior Prob. CAR <SPDE	CAR model	SPDE model
Brier	0.773	0.765	0.10	0.710	0.710
Negative Log Density	355928	353987	0.25	311525	311902
Mean Absolute Error	0.0309	0.0296	0.10	0.0226	0.0223
Root Mean Square Error	0.0763	0.0739	0.02	0.0533	0.0530

Smaller values are better for all metrics.

**Table 4:** Coverage and length of prediction intervals for the CAR and SPDE spatial models when holding out 80% of entire cells of data in Minnesota.

	CAR model	SPDE model
Coverage	0.981	0.972
Mean Interval Length	0.112	0.103
Median Interval Length	0.028	0.022

Coverage values near 0.95 are optimal, while shorter intervals are better.

In Experiment 2 (individual trees held out), we have evidence (posterior probability of 0.93) that the SPDE model is better based on the Brier score, but the Brier score values for the two models are numerically almost the same (Table 5).

**Table 5:** Predictive ability based on several predictive metric criteria for the CAR and SPDE spatial models when holding out 5% of trees.

	Posterior mean of metric			Metric of posterior mean predictions	
	CAR model	SPDE model	Posterior Prob. CAR <SPDE	CAR model	SPDE model
Brier	0.662	0.661	0.07	0.657	0.657
Negative Log Density	51757	51626	0.01	50705	50736

Smaller values are better for all metrics.

The differences between models are not consistent across the various comparisons, so there is not a clear choice. In our final data product we use the CAR model, for three reasons. First, the CAR model has modestly better performance when data are sparse, as is still the case for Illinois and Indiana. Second, the model is simpler and easier to explain, and computations can be done more quickly. Third, predictions from the SPDE model showed boundary effects, with some taxa showing non-negligible posterior mean values at the edges of the domain, well away from where the taxa were present in the empirical data. This included non-negligible values within (but near the edge of) the convex hull of locations with data.

## Data Product

The final data product is a dataset that contains 250 posterior samples of the proportions of each of the 23 tree taxa at each grid cell in the states in our domain of the northeastern United States.

For this final data product, we ran the model using the CAR specification with all of the data (including the data held out in the model comparison analyses) for 150,000 iterations with the same burn-in and subsampling details as described in *Model comparison*. Based on graphical checks and calculation of effective sample size values, mixing was generally reasonable, but for some of the hyperparameters was relatively slow, particularly for less common taxa. Despite this, mixing for the variables of substantive interest—the proportions—was good, with effective sample sizes for the final product generally near 250.

**Fig 2:**
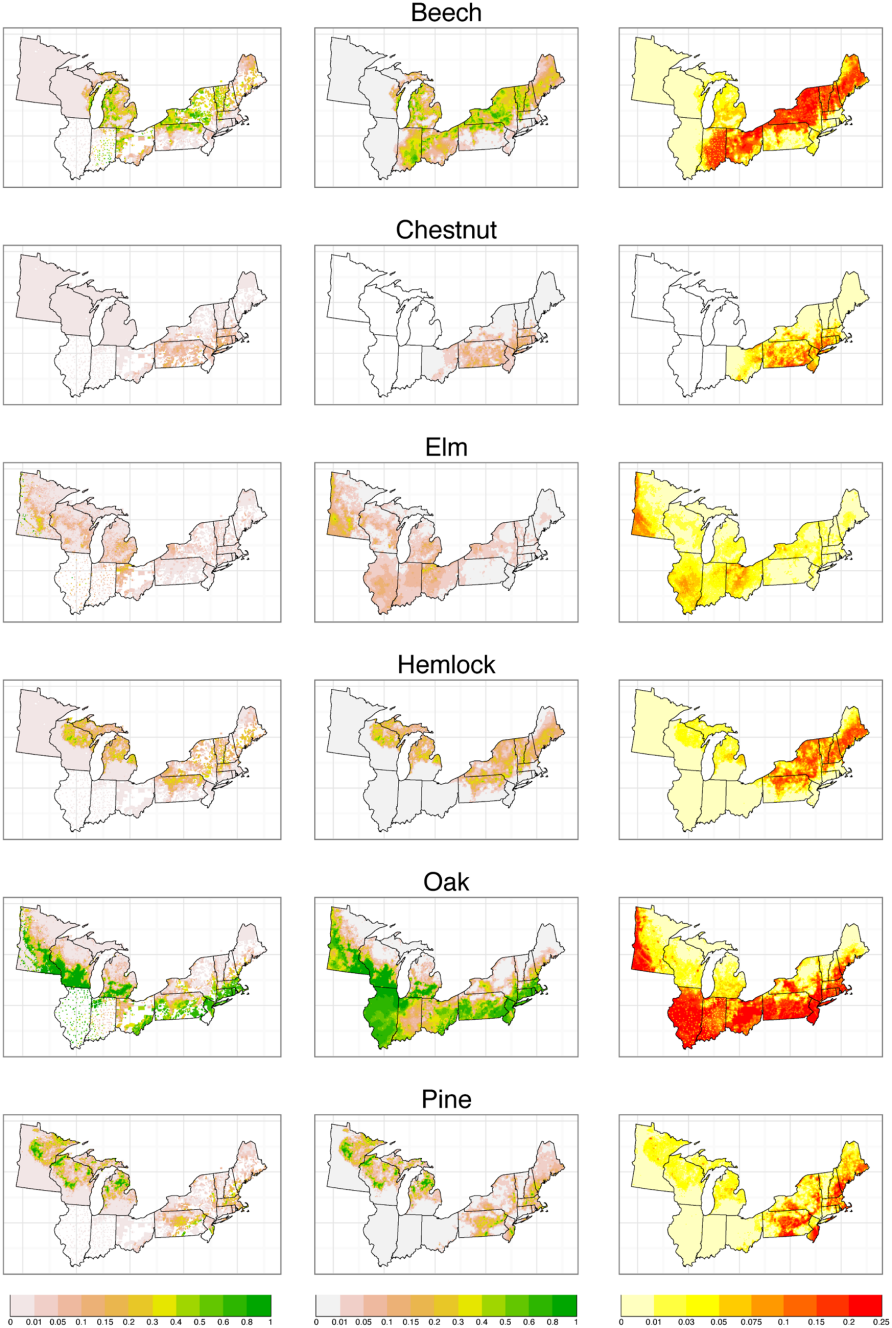
Raw data, predictions, and uncertainty for select taxa. Empirical proportions from raw data (column 1), predictions in the form of posterior means (column 2) and uncertainty estimates in the form of posterior standard deviations—representing standard errors of prediction (column 3). In raw data plots, white indicates no data.

**Fig 3:**
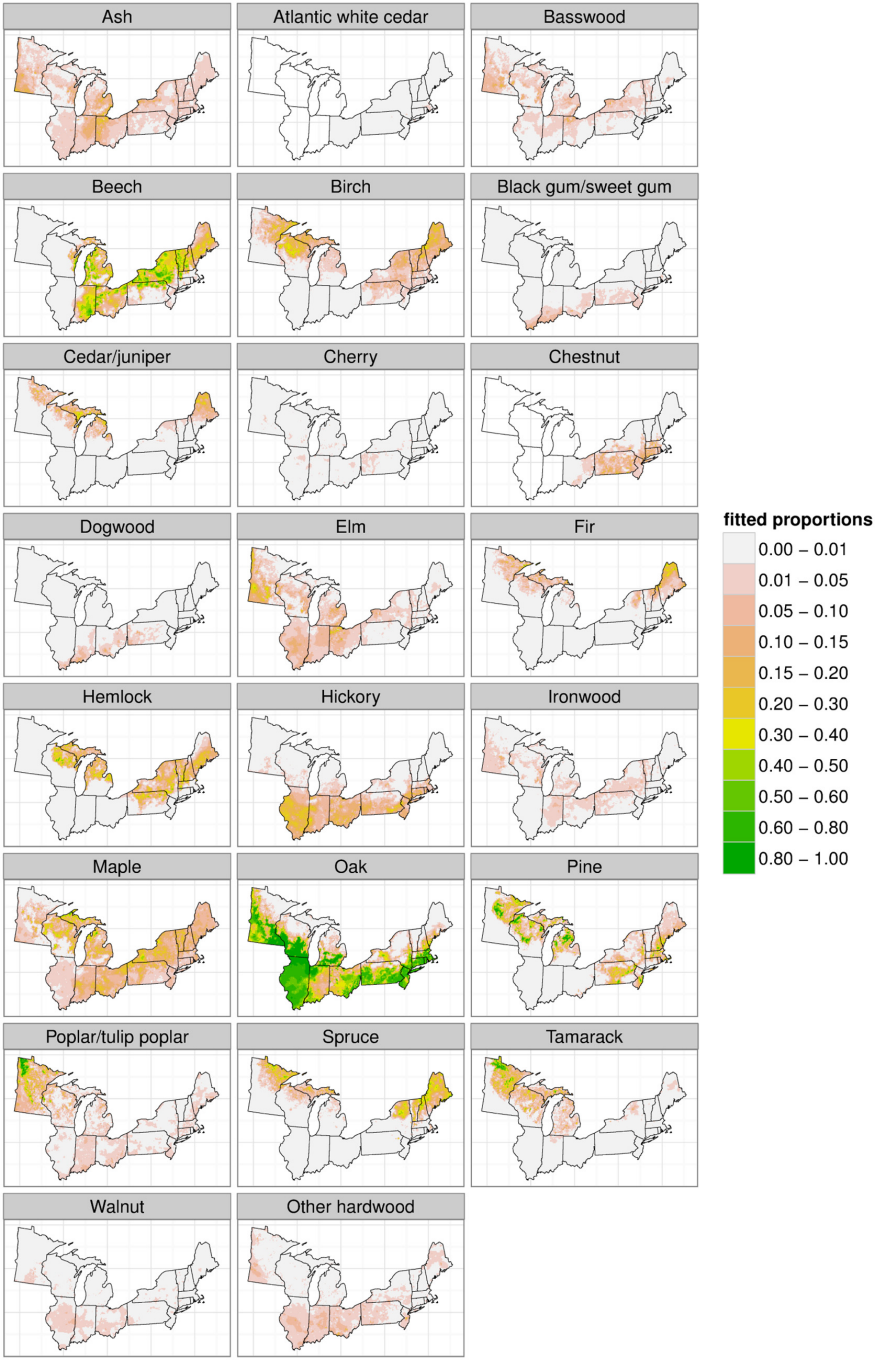
Predictions (posterior means) for all taxa over the entire domain.

Maps of estimated composition for the full domain for several taxa of substantive interest illustrate the results, contrasting the raw data proportions, the posterior means, and posterior standard deviations as pointwise estimates of uncertainty (Fig 2). We also present the posterior means for all 23 taxa (Fig 3).

The data product is publicly available at the NIS Data Portal under the CC BY 4.0 license as version 1.0 as of February 2016 [34]. The product is in the form of a netCDF-4 file, with dimensions x-coordinate, y-coordinate, and MCMC iteration. There is one variable per taxon. In addition, dynamic visualizations of the product using the Shiny tool are available at https://www3.nd.edu/~paleolab/paleonproject/maps. The PalEON Project (in particular the first author) will continue to maintain this product, releasing new versions as additional data in Illinois, Indiana and Ohio are digitized. Note that digitization of data from Illinois and Indiana is ongoing, and digitization of additional data from Ohio is planned as well. As a result, at some point we expect to have complete data for the midwestern half of the domain.

## Discussion

In the parts of the modeled region with spatially complete data (in particular, Minnesota, Wisconsin, and Michigan), the statistical estimates of forest composition closely match the patterns apparent in the raw data (Fig 2), as expected. In these areas, the estimated tree composition from the model has the advantage of downweighting unusual or outlier values in the empirical proportions of individual grid cells, which are likely due to stochastic sampling variation within that grid cell (compare the first two columns in Fig 2). Some stochastic variation is expected given that, even in the most spatially complete regions, each grid cell contains an average of 124 trees (120-135 is typical) [6] and some cells contain many fewer trees. Hence, some smoothing of this stochastic variation is appropriate. This smoothing is based on information on data from nearby cells, and the estimates from the model reflect the smooth trends in forest composition across the spatial domain. A partial cost is that these maps can smooth out sharp ecotones or other forms of true spatial heterogeneity, particularly in areas with sparse data (including areas with low tree density). For example, the sharp increase in Elm along the Minnesota River (Fig 2, first column) likely represents a real ecotone in the settlement-era vegetation. Vegetation gradients and ecotones were sharper in the settlement-era forests in the upper Midwest than in contemporary forests [6], and the modeled estimates may partially obscure this change. Users interested in using the original unsmoothed data are directed to the data product described earlier [17]. Additional investigation of other statistical representations to better capture sharp gradients is of interest, in particular nonstationary spatial models and use of covariates. Potential environmental covariates include soils, firebreaks, and topography [35, 36]. Here, however, we chose to limit our model to be exclusively a function of spatial distance in order to avoid dependence on the environmental drivers of pre-settlement forest composition that might lead to circular reasoning in subsequent inferences based on this dataset. Use of covariates could also lead to prediction that a taxa is present well beyond its range boundary in places where data are sparse.

A key advance of this work over prior reconstructions of settlement-era vegetation lies in the estimates of uncertainty across the spatial domain. These estimates of uncertainty include the sampling uncertainty within grid cells (as do the within-grid cell estimates of uncertainty available from the raw proportions), but, because this is a spatial model, predictions and their associated uncertainty estimates are also informed by the information content of nearby cells. The maps of standard errors across species (Fig 2, third column) highlight the advantages of this approach in areas of high data coverage (Minnesota, Wisconsin, Michigan) and in areas of sparse coverage (e.g., Illinois, Indiana, parts of Ohio). Where there are not large gaps in the data, the model provides low and fairly smooth estimates of uncertainty. Uncertainty is generally higher in the eastern subdomain than in the areas of the midwestern subdomain with high data coverage because of missing townships and lower sampling density even in townships with data. In areas of sparse coverage and in areas with low tree density (e.g., southwestern Minnesota), the standard error of our estimates increases appropriately. Nevetheless, these uncertainties surround reasonable estimates of trends in composition. For example, the model does a good job of capturing the oak ecotone in Indiana and Illinois, representing a shift from oak savannas and woodlands to closed mesic forests (Fig 2). Experiment 1 showed that both models predicted composition at cells with no data reasonably well, mimicking the case of sparsely sampled data and giving confidence in the broad spatial patterns predicted in more poorly sampled regions, particularly those with regular, but sparse sampling that mimic the experiment (Illinois and Indiana, but not Ohio). The apparent blockiness of uncertainty estimates in a few places such as Ohio is caused by spatial gaps and variations in sampling resolution. Absolute uncertainty generally increases with abundance for all taxa (Fig 2, column 3).

The exploration of alternative approaches to spatial modeling of composition showed similar results for the SPDE and CAR models, both in terms of prediction accuracy and performance of prediction intervals. Small differences among the various metrics of goodness of fit favored each model in turn, but applied users of the models would find little pragmatic difference affecting scientific inference. Ultimately, we slightly favor the CAR model, because it avoids the boundary effects apparent in the SPDE model at the edges of the domain.

The models presented here estimate only the relative abundance of tree taxa, which does not directly tell us about tree density or other aspects of vegetation structure. This becomes a particular limitation for interpreting vegetation where trees become sparse at the prairie-forest transition from northern Minnesota through southern Illinois [37]. Our model (correctly) predicts that tree composition there is dominated by oak, but this is less useful considering the sparseness of trees. This limitation can be addressed by developing estimates of absolute abundance (e.g., biomass) rather than compositional estimates. A gridded dataset of biomass, stem density, and basal area is already available for Minnesota, Wisconsin, and northern Michigan [6], based on the PLS data. An extension to southern Michigan, Illinois, and Indiana is planned. We are currently developing statistical estimates of biomass for Minnesota, Wisconsin, and Michigan using a statistical model applied to the gridded biomass dataset, with extension to Illinois and Indiana planned. We also plan to estimate stem density and basal area using a similar approach to that used for biomass.

## Appendix

### MCMC details

Define ${\bar{w}}_{ip}=\frac{1}{n_{i}}\sum_{j=1}^{n_{i}}W_{ijp}$ as the average of the *W* values for the *p*th taxon in the *i*th grid cell and ${\bar{w}}_{p}=\left\{{\bar{w}}_{ip}\right\},$ *i* = 1, …, *m*. Let *A* be a diagonal matrix where *A*_*ii*_ is the number of trees in the *i*th grid cell. When there are no trees in a grid cell, ${\bar{w}}_{ip}=0$ and *A*_*ii*_ = 0. For the township data, at each iteration, based on the current values of the grid cell membership variables, {*c*_*tj*_}, trees are aggregated into grid cells and the calculations above can then be carried out.

The conditional distribution for *W*_*ijp*_ given the other unknowns in the model and the data is as follows. Let TN(*a*, *b*, *μ*, *τ*^2^) denote the truncated normal distribution with mean parameter *μ* and variance parameter *τ*^2^, truncated below by *a* and above by *b*.

$$\begin{array}{c} W_{ijp}∼\{\begin{array}{cc} \text{TN}(\max_{p^{*}\neq y_{ij}}w_{ijp^{*}},\infty ,\alpha_{y_{ij}}\left(s_{i}\right),1), & \text{if}\;p=y_{ij}\\ \text{TN}(-\infty ,w_{ijy_{ij}},\alpha_{p}\left(s_{i}\right),1), & \text{if}\;p\neq y_{ij} \end{array} \end{array}$$

In essence, the truncation value is determined by the taxon of the *j*th tree. For a given *p*, the *W* values for all trees in all cells can be sampled in parallel.

The conditional distribution of *α*_*p*_ is

$$\begin{array}{c} \alpha_{p}∼N({\left(A+Q_{p}\right)}^{-1}A{\bar{w}}_{p},{\left(A+Q_{p}\right)}^{-1}). \end{array}$$

where $Q_{p}={\left(\sigma_{p}^{2}\right)}^{-1}Q$ for the CAR model and ${\left(\sigma_{p}^{2}\cdot \frac{4\pi }{\rho_{p}^{2}}\right)}^{-1}Q\left(\rho_{p}\right)$ for the SPDE model. For each hyperparameter, *ϕ*_*p*_ = log *σ*_*p*_ for the CAR model and *ϕ*_*p*_ ∈ {*μ*_*p*_, (log *σ*_*p*_, log *ρ*_*p*_)} for the SPDE model, we sample {*ϕ*_*p*_, *α*_*p*_} jointly, proposing *ϕ*_*p*_ as a random walk and, conditional on the proposed value of *ϕ*_*p*_, sampling *α*_*p*_ from the distribution just above. The joint proposal is accepted or rejected as a standard Metropolis-Hastings proposal, with adaptation of the proposal (co)variance [28]. The proposal distribution for *ϕ*_*p*_ is a normal distribution (bivariate for *ϕ*_*p*_ = (log *σ*_*p*_, log *ρ*_*p*_)).

For the township-level data, for a given tree *j* in township *t*, we draw the latent tree membership variable, *c*_*tj*_ ∈ {1, …, *m*}, from a discrete distribution by normalizing posterior weights, {*ψ*_1_ *L*_*tj*1_, …, *ψ*_*m*_ *L*_*tjm*_}, produced by multiplying the prior weights by a likelihood contribution, *L*_*tji*_, *i* = 1, …, *m*. *L*_*tji*_ is the density of the latent *W*_*tj*1_, …, *W*_*tjP*_ values for the given tree under the condition that *c*_*tj*_ = *i*, namely the product of independent normal densities, $W_{tjp}∼N\left(\alpha_{p}\left(s_{i}\right),1\right)$, over *p* = 1, …, *P*. Thus the posterior reweights the prior based on how consistent the current *W*_*tj*_ values for a tree are with the *α* values for the candidate grid cells.

### Estimating *θ*_*p*_(*s*) via Monte Carlo integration

In the latent variable representation, *θ*_*p*_(*s*) never appears explicitly and cannot be calculated in closed form. Instead we use Monte Carlo integration over *W*_*ijp*_, *p* = 1, …, *P* to estimate *θ*_*p*_(*s*_*i*_). The quantity $\theta_{p}\left(s_{i}\right)=\text{Prob}(W_{ijp}=\max_{p^{*}}W_{ijp^{*}})$ defines the probability of taxon *p* at grid cell *i*. This requires one to choose the number of Monte Carlo samples, which we set at 10000, effectively sampling 10000 hypothetical trees and estimating the probabilities of the different taxa in the population from the empirical proportions in this sample of trees. For each of the saved MCMC samples, *k* = 1, …*K*, we estimate $\theta_{p}^{\left(k\right)}\left(s_{i}\right)$ numerically. Specifically, for *t* = 1, …, 10000 samples (i.e., hypothetical trees), we independently draw

$$\begin{array}{c} W_{itp}^{\left(k\right)}∼N\left(\alpha_{p}^{\left(k\right)}\left(s_{i}\right),1\right),\;p=1,\ldots ,P \end{array}$$

and estimate using

$$\begin{array}{c} \theta_{p}^{\left(k\right)}\left(s_{i}\right)\approx \frac{1}{10000}\sum_{t=1}^{10000}1(W_{itp}^{\left(k\right)}=\max_{p^{*}}W_{itp^{*}}^{\left(k\right)}),\;p=1,\ldots ,P \end{array}$$

where 1(⋅) is the indicator function that evaluates to 1 if the expression is true and 0 if false. In other words, we calculate the proportion of times that the maximum of *W*_*itp*_, *p* = 1, …, *P* corresponds to taxon *p*. Considering $\theta_{p}^{\left(k\right)}\left(s_{i}\right),\;k=1,\ldots ,K$, we have a sample from the posterior of *θ*_*p*_(*s*_*i*_).
