# Correction: Statistically-Estimated Tree Composition for the Northeastern United States at Euro-American Settlement

**DOI:** 10.1371/journal.pone.0170835

**Published:** 2017-01-20

**Authors:** Christopher J. Paciorek, Simon J. Goring, Andrew L. Thurman, Charles V. Cogbill, John W. Williams, David J. Mladenoff, Jody A. Peters, Jun Zhu, Jason S. McLachlan

The Data Availability statement for this paper is incorrect. The correct statement is: The final data product is available through the NIS data portal (https://portal.lternet.edu) as DOI:10.6073/pasta/8544e091b64db26fdbbbafd0699fa4f9. The gridded input data at the 8 km resolution for the midwest subdomain are available through the NIS data portal as DOI:10.6073/pasta/aa0ef9828d41569a96651b056ad89fb3. The township input data for the eastern subdomain are available through the NIS data portal as DOI:10.6073/pasta/e40c1ad172882d5113844296611451f6 and DOI:10.6073/pasta/d09bb56c6af8ef0783cd7e76f8113b34.

There is an error in reference 19. The correct reference is: Cogbill CV. Settlement-era tree composition, Ohio: level 1. Long Term Ecological Research Network; 2016. http://dx.doi.org/10.6073/pasta/d09bb56c6af8ef0783cd7e76f8113b34.
